# The relationship between compassion fatigue and perceptions of spirituality and spiritual care in intensive care unit nurses: a cross-sectional study

**DOI:** 10.1186/s12912-026-04630-y

**Published:** 2026-04-18

**Authors:** Adile Neşe, Belgin Yiğit, Ercan Bakır

**Affiliations:** 1 Vocational School of Health Services, Gaziantep University, Gaziantep, Turkey; 2 Faculty of Health Sciences Department, Adıyaman University, Adıyaman, Turkey

**Keywords:** Intensive care nurses, Compassion fatigue, Spirituality, Spiritual care

## Abstract

**Background:**

Intensive care units (ICUs) are high-stress environments that significantly predispose nurses to compassion fatigue, affecting both their well-being and the quality of patient care. Spirituality and spiritual care are often considered potential coping mechanisms; however, their specific relationship with compassion fatigue remains under-explored in the ICU context.

**Aim:**

This study aimed to investigate the relationship between compassion fatigue and the perceptions of spirituality and spiritual care among nurses working in intensive care units.

**Study design:**

This descriptive, correlational, and cross-sectional study was conducted between March 15 and April 30, 2023, in all intensive care units of Gaziantep University Research and Practice Hospital in southeastern Türkiye. A non-probability total population sampling method was used, targeting all ICU nurses at the facility. The final sample consisted of 122 nurses who participated voluntarily. Data were collected using the Introductory Information Form, the Short-Form Compassion Fatigue Assessment, and the Spiritual Care and Spirituality Perception Instrument. Data were analyzed using descriptive and inferential statistical methods in SPSS 25.0 (IBM, New York, USA).

**Results:**

The mean age of the participating nurses was 30.49±6.30 years, and their mean duration of work in the intensive care units was 5.24±4.18 years. The mean total score of the Compassion Fatigue Assessment was 62.63±29.41, while the mean total score on the Spiritual Care and Spirituality Perception Instrument was 42.33±6.87. No significant relationship was found between compassion fatigue and the total score of the Spiritual Care and Spirituality Perception Instrument or most of its subscales. However, a weak but statistically significant positive correlation was identified only between compassion fatigue and the religiosity subscale (r=0.20).

**Conclusion:**

This study did not demonstrate a significant relationship between compassion fatigue and overall spirituality and spiritual care perception. Instead, the only significant but weak association was observed between compassion fatigue and the religiosity subscale. These findings suggest that while religiosity may be related to compassion fatigue, overall spirituality and spiritual care perception is not significantly associated with compassion fatigue in intensive care nurses.

**Clinical trial number:**

Not applicable.

## Introduction

Nurses are essential health professionals who play a critical role in providing basic health services and frequently encounter patients’ pain, anxiety, hopelessness, and traumatic experiences [1]. These challenges become even more pronounced in intensive care units, where patients require close monitoring due to changes in vital functions and life-threatening conditions that may lead to suffering for patients, their families, and healthcare staff [2, 3]. The primary responsibility of intensive care nurses is to deliver appropriate and ethical care, prevent life-threatening complications, and ensure the best possible outcomes for patients [2].

Compassion fatigue is a concept that describes the diminished capacity or interest to care, emerging from prolonged exposure to patients’ suffering and traumatic events. It is characterized by emotional, physical, and spiritual exhaustion that negatively affects nurses’ professional well-being [4, 5]. Due to the demanding nature of intensive care settings, nurses working in these units are at heightened risk of developing compassion fatigue. Factors such as heavy workload, unexpected patient deaths, communication difficulties with families, high performance expectations, time pressure, and limited social support contribute significantly to stress and reduced job satisfaction [6, 7].

The consequences of compassion fatigue are substantial, influencing both professional functioning and personal well-being. Intensive care nurses experiencing compassion fatigue may demonstrate impaired job performance, decreased productivity, increased turnover intention, and psychosocial difficulties, all of which negatively impact the quality of patient care [8–10]. The complex care needs and frequent exposure to human suffering inherent in intensive care units further amplify these risks [8]. Within this context, spiritual care represents an essential component of holistic nursing practice, particularly in intensive care environments where patients face uncertainty, fear, and existential distress. Spiritual care has been shown to enhance comfort and spiritual well-being, reduce anxiety and helplessness, and support coping with illness [11]. It also contributes to improved hope, life satisfaction, care quality, and patient satisfaction [12–14]. Nurses’ ability to provide effective spiritual care largely depends on their understanding of spirituality and sensitivity to patients’ spiritual needs, influenced by personal beliefs, communication skills, work environment, and interactions with healthcare teams [12, 15]. Despite the recognized importance of both compassion fatigue and spiritual care in intensive care settings, the relationship between these two concepts remains insufficiently understood. Limited evidence exists regarding how compassion fatigue may influence nurses’ perceptions of spirituality and spiritual care. Given the high psychological and spiritual demands of intensive care and the culturally significant role of spirituality in Turkey, investigating this relationship is crucial for enhancing holistic nursing practice and informing targeted support strategies. This gap in the literature highlights the need for studies that explore the interplay between compassion fatigue and spiritual care perceptions among intensive care nurses.

### Aim of the study

The aim of this study was to investigate the relationship between compassion fatigue and perceptions of spirituality and spiritual care including the subdimensions of religiosity and individualized care among nurses working in intensive care units in Turkey. By assessing the levels of these variables and their interplay, this study seeks to provide evidence based insights to support holistic nursing practice and inform targeted psychological support strategies.

### Research hypothesis

#### H1

There is a statistically significant relationship between compassion fatigue and nurses’ perceptions of spirituality and spiritual care among intensive care unit nurses.

## Methods

This study employed a descriptive, correlational, and cross-sectional design. This study employed a descriptive, cross-sectional, and correlational design. This approach was selected to simultaneously determine the levels of compassion fatigue and spirituality/spiritual care perceptions (primary aim) while examining the correlational relationships between these variables and their subdimensions (secondary aim) among intensive care nurses. The study was conducted between March 15 and April 30, 2023, at Gaziantep University Research and Practice Hospital in southeastern Türkiye. Data were collected from six specialized intensive care units (ICUs), specifically: Internal Medicine, Neurosurgery, Neurology, Coronary and Cardiovascular, Anesthesia, and Pediatric ICUs.

### Population and sample

The study population consisted of 152 nurses. A total population sampling method was employed, aiming to reach every eligible nurse within the specified units. To ensure a representative sample of the regular ICU workforce, inclusion criteria were: (1) having at least one year of professional experience in an ICU setting and (2) voluntary agreement to participate. Exclusion criteria included: nurses on extended leave, those with known active mental health disorders, or those currently in temporary or part-time rotations. Of the 152 eligible nurses, 122 nurses voluntarily agreed to participate and completed the study questionnaires, resulting in a response rate of 80.3%.

Considering the critical and demanding environment of intensive care units, survey forms were distributed electronically. Although WhatsApp was used to distribute surveys prepared on Google Forms to facilitate participation and increase response rates, potential limitations such as selection bias and lack of controlled data collection were acknowledged. To mitigate these issues, all eligible nurses were invited to participate, clear instructions were provided, and responses were monitored for completeness and consistency. Data were collected only from nurses who provided informed consent, and all procedures adhered to ethical standards. Nurses who had at least one year of experience in intensive care units and voluntarily agreed to participate were included in the study. Nurses working in departments other than intensive care units, those who declined participation, or those on leave (e.g., stress leave, medical leave), with known mental disorders, or engaged in part-time or temporary rotations were excluded. These criteria were applied to ensure that participants were representative of the regular ICU nursing workforce and capable of completing the study questionnaires reliably.

An a priori sample size calculation was not conducted, as the study was designed to include the entire accessible population. However, a post-hoc power analysis was performed to evaluate the adequacy of the final sample size for correlation analysis. Based on a moderate effect size (*r* = 0.30), a significance level of α = 0.05, and the achieved sample size (*n* = 122), the statistical power was calculated to be greater than 80%, indicating that the sample size was sufficient to detect meaningful correlations.

### Data collection tools

The data for the research were collected using the Introductory Information Form, the Compassion Scale Short Form, and the Spiritual Care and Spirituality Perception Instrument.

### Introductory information form

It consists of questions prepared by the researchers including the socio-demographic characteristics of the participants. In addition, these selected variables (age, gender, education, years of employment, intensive care unit where they worked) were determined by citing similar studies in the literatüre [1–3, 6].

### Short form compassion fatigue assessment (SF-CFA)

Developed by Adams et al. (2006) to evaluate compassion fatigue in healthcare workers, the form evaluates compassion fatigue in 13 items [16]. The validity and reliability study of the scale in Turkey was conducted by Dinç and Ekinci (2019). Each item is rated on a Likert-type scale, with total scores ranging from 13 to 130. Higher scores indicate higher levels of compassion fatigue experienced by the individual. The SF-CFA includes two subdimensions: burnout and secondary traumatic stress. The validity and reliability of the scale in Turkey were established by Dinç and Ekinci (2019), who reported a Cronbach’s alpha coefficient of 0.87 for the total scale [10]. In the present study, the Cronbach’s alpha for the total scale was 0.80, confirming adequate internal consistency.

### Spiritual care and spirituality perception instrument (SCSPI)

The Spirituality and Spiritual Care Perception Instrument (SCSPI), developed by McSherry et al. (2002), is a 17-item, five-point Likert-type scale designed to measure nurses’ spirituality and spiritual care perceptions [17]. Scores range from 17 to 85, with higher scores indicating stronger perceived spirituality or quality of spiritual care. The scale comprises four subscales: spirituality, spiritual care, religiosity, and personalized care. The SCSPI was validated in Turkey by Ergül and Temel (2007), who reported a Cronbach’s alpha of 0.76 for the total scale and satisfactory subscale reliabilities [18]. In the present study, the Cronbach’s alpha for the total scale was 0.71, indicating acceptable internal consistency.

### Data analysis

The analyses were conducted using the SPSS version 25.0 (IBM Corp., New York, USA). The Shapiro–Wilk test and Levene’s test were used to assess the normality of distribution and homogeneity of variances, respectively. Descriptive statistics, including percentages, means, and standard deviations, were utilized to summarize the data. For the comparison of continuous variables between two independent groups, the Independent Samples t-test was used for normally distributed data, while the Mann–Whitney U test was applied for non-parametric data. For comparisons involving three or more groups, One-Way Analysis of Variance (ANOVA) was performed when assumptions were met, followed by Tukey’s Honestly Significant Difference (HSD) post-hoc test to identify specific group differences. In cases where the normality assumption was violated, the Kruskal–Wallis test was employed, with post-hoc pairwise comparisons conducted using Dunn’s test involving Bonferroni correction. To examine the relationships between continuous variables, Pearson or Spearman correlation coefficients (r) were calculated based on the distribution. The internal consistency of the scales was evaluated using Cronbach’s alpha. Statistical significance was set at *p* < 0.05, with a 95% confidence interval.

### Ethics statement

The study was conducted in accordance with the Declaration of Helsinki. Ethical approval was obtained from the Gaziantep University Ethics Committee (Date: 21 December 2022; Decision No: 2022/424) and institutional permission was secured. Written informed consent was obtained electronically via Google Forms. Participants confirmed consent by selecting “I agree to participate” before accessing the questionnaire. All data and consent records were stored securely in password-protected files, with no personal identifiers collected. Participation was voluntary, and withdrawal was allowed at any time.

## Results

The mean age of the intensive care nurses was 30.49 ± 6.30 years, with an average ICU experience of 5.24 ± 4.18 years. Most were aged 20–29 (53.3%), female (77.9%), married (61.5%), and undergraduates (63.1%). They worked mainly in brain/surgical (22.2%) and internal medicine ICUs (18.0%),and 72.1% working night shifts. The distribution of professional experience showed that 52.5% of the nurses had 1–4 years of ICU experience. This cluster is consistent with the predominant 20–29 age group and undergraduate education level, reflecting a workforce composed largely of early-career professionals who entered the intensive care unit shortly after graduation. Regarding job satisfaction, 34.4% were satisfied, 47.5% sometimes satisfied; 41.0% sometimes considered transferring, 46.7% did not consider leaving, and 14.8% wanted to leave. CFA were significantly associated with women, nurses with postgraduate education, those working in neurology ICUs, rarely satisfied staff, those considering resignation, and those strongly desiring a transfer (*p*<0.05). Age, marital status, ICU experience, and work schedule had no significant associated with CFA scores (*p*>0.05). These findings indicate that certain demographic and professional factors show associations with different levels of compassion fatigue among nurses (Table 1).

**Table 1:** Distribution of Total Compassion Fatigue Assessment (CFA) Scores by Socio-Demographic Characteristics of Intensive Care Nurses (*n* = 122)

X ± SD	*n* (%)	CFA Mean ± SD	Significance Test
		62.63 ± 29.41	
**Age**			
20–29 years	65 (53.3%)	58.72 ± 4.74	F = 1.568, *p* = 0.213
30–39 years	43 (35.2%)	68.88 ± 32.01	
40–49 years	14 (11.5%)	61.64 ± 20.09	
**Gender**			
Female	95 (77.9%)	66.50 ± 30.05	t = 2.799, *p* = 0.007
Male	27 (22.1%)	49.03 ± 22.68	
**Marital Status**			
Married	75 (61.5%)	67.18 ± 29.26	t = 2.191, *p* = 0.838
Single	47 (38.5%)	55.38 ± 28.46	
**Education Level**			
High School	13 (10.7%)	53.46 ± 31.14	F = 4.548, *p* = 0.005
Associate Degree	20 (16.4%)	44.55 ± 24.51	
Bachelor’s Degree	77 (63.1%)	67.01 ± 27.20	
Postgraduate	12 (9.8%)	74.66 ± 36.35	
**ICU Unit Worked In**			
Internal Medicine ICU	22 (18.0%)	64.00 ± 31.16	F = 3.053, *p* = 0.013
Neurosurgery ICU	27 (22.2%)	49.25 ± 27.86	
Neurology ICU	12 (9.8%)	78.25 ± 30.78	
Coronary and Cardiovascular ICU	11 (9.0%)	63.90 ± 23.85	
Anesthesia ICU	19 (15.6%)	53.78 ± 22.85	
Pediatric ICU	31 (25.4%)	72.25 ± 29.66	
**Duration of ICU Experience**			
1–4 years	64 (52.5%)	57.73 ± 29.08	F = 2.281, *p* = 0.107
5–10 years	42 (34.4%)	66.02 ± 29.23	
11–15 years	16 (13.1%)	73.37 ± 28.83	
**Happiness in ICU**			
Rarely happy	22 (18.1%)	85.77 ± 27.24	F = 20.323, *p* = 0.000
Sometimes happy	58 (47.5%)	66.98 ± 25.62	
Very often happy	42 (34.4%)	44.52 ± 24.70	
**Desire to Transfer**			
Rarely	42 (34.4%)	48.78 ± 23.89	F = 27.115, *p* = 0.000
Sometimes	50 (41.0%)	57.52 ± 26.74	
Very often	30 (24.6%)	90.56 ± 21.53	
**Considering Leaving Job**			
Yes	18 (14.8%)	82.00 ± 28.77	F= -10.598, *p* = 0.000
Sometimes	47 (38.5%)	68.89 ± 26.89	
No	57 (46.7%)	51.36 ± 27.22	
**Work Schedule**			
Day shift	34 (27.9%)	70.14 ± 28.50	t = 1.775, *p* = 0.078
Night shift	88 (72.1%)	59.72 ± 29.39	

F: ANOVA Test, t: Student t Test, *p*<0.05

Higher SCSPI subscale and total scores were observed among women, single nurses, those aged 20–29, high school graduates, internal medicine ICU staff, those rarely satisfied with their unit, frequently considering transfer, sometimes considering leaving their job, and day-shift workers. Significant differences appeared between gender and desire to transfer with the religiosity subscale, and between unit satisfaction with the spiritual care subscale and total SCSPI scores (*p* < 0.05). No significant differences were found for age, marital status, education, ICU type, intention to leave, or shifts (*p* > 0.05) (Table 2). The mean total score of the CFA was 62.63 ± 29.41, indicating a moderate level of compassion fatigue among intensive care nurses when evaluated according to the scale’s score range. Spirituality and Spiritual Care Perception Inventory was 42.33 ± 6.87, suggesting a moderate perception of spirituality and spiritual care. Subscale mean scores were 14.44 ± 3.89 for spirituality and spiritual care, 8.93 ± 2.46 for individualized care, and 14.40 ± 2.49 for individual religiosity.

**Table 2:** Distribution of average total scores of SCSPI and subscales based on socio-demographic characteristics of intensive care unit nurses (*n* = 122)

Variable		*n*	Spiritual care	Individual care	Religiosity	SCPSS
X ± SD			14.44 ± 3.89	8.93 ± 2.46	14.40 ± 2.49	42.33 ± 6.87
Age	20–29	65	15.07 ± 4.09	9.09 ± 2.65	14.16 ± 2.73	38.33 ± 7.13
	30–39	43	13.95 ± 3.86	8.76 ± 2.34	14.41 ± 2.30	37.13 ± 6.16
	40–49	14	13.00 ± 2.28	8.71 ± 1.97	15.42 ± 1.60	37.14 ± 3.41
	F/p		2.206/0.115	0.284/0.753	1.480/0.232	0.518/0.590
Gender	Female	95	14.48 ± 3.95	9.03 ± 2.51	14.83 ± 2.30	38.34 ± 6.36
	Male	27	14.29 ± 3.72	8.59 ± 2.30	12.88 ± 2.57	35.77 ± 6.51
	t/p		0.220/0.826	0.815/0.417	**3.759/0.000**	1.842/0.068
Marital Status	Married	75	14.06 ± 3.56	8.94 ± 2.33	14.60 ± 2.24	37.61 ± 5.74
	Single	47	15.04 ± 4.33	8.91 ± 2.69	14.08 ± 2.84	38.04 ± 7.52
	t/p		-1.352/0.179	0.069/0.945	1.110/0.269	-0.356/0.723
Education Level	High School	13	15.92 ± 6.13	10.00 ± 4.14	13.61 ± 4.15	39.53 ± 12.84
	Associate Degree	20	14.25 ± 3.72	8.75 ± 2.07	14.05 ± 2.28	37.05 ± 5.68
	Bachelor’s Degree	77	14.57 ± 3.38	8.81 ± 2.18	14.42 ± 2.31	37.81 ± 5.09
	Postgraduate	12	12.33 ± 3.84	8.83 ± 2.51	15.66 ± 0.98	36.83 ± 6.19
	F/p		1.886/0.136	0.909/0.439	1.618/0.189	0.487/0.692
ICU Unit Worked In	Internal Medicine	22	16.00 ± 4.27	9.27 ± 2.09	14.22 ± 2.11	39.50 ± 5.84
	Neurosurgery	27	14.18 ± 3.31	8.66 ± 2.35	14.22 ± 2.00	37.07 ± 5.09
	Neurology	12	13.16 ± 4.10	8.75 ± 2.34	15.16 ± 2.44	37.08 ± 5.07
	Coronary and Cardiovascular	11	13.09 ± 1.92	7.81 ± 1.94	13.18 ± 3.06	34.09 ± 4.92
	Anesthesia	19	15.47 ± 4.97	9.78 ± 2.93	14.63 ± 2.77	39.89 ± 8.69
	Pediatric	31	13.90 ± 3.52	8.87 ± 2.66	14.67 ± 2.76	37.45 ± 6.94
	F/p		1.682/0.144	1.075/0.378	0.905/0.480	1.582/0.170
Duration of ICU Experience	1–4 years	64	14.79 ± 3.59	8.65 ± 2.14	14.35 ± 2.44	37.81 ± 5.43
	5–10 years	42	14.38 ± 4.50	9.16 ± 2.97	14.35 ± 2.86	37.90 ± 8.38
	11–15 years	16	13.18 ± 3.16	9.43 ± 2.15	14.68 ± 1.62	37.31 ± 4.45
	F/p		1.104/0.335	0.926/0.399	0.119/0.888	0.050/0.951
Happiness in ICU	Rarely happy	22	17.09 ± 3.22	9.68 ± 2.25	14.04 ± 2.71	40.81 ± 5.15
	Sometimes happy	58	14.48 ± 4.03	9.06 ± 2.66	14.93 ± 2.11	38.48 ± 6.51
	Very often happy	42	13.00 ± 3.29	8.35 ± 2.18	13.85 ± 2.75	35.21 ± 6.17
	F/p		**9.035/0.000**	2.297/0.105	2.596/0.079	**6.659/0.002**
Desire to Transfer	Rarely	42	13.97 ± 3.93	8.71 ± 2.23	13.50 ± 2.75	36.19 ± 6.98
	Sometimes	50	14.38 ± 3.77	8.78 ± 2.59	14.82 ± 2.27	37.98 ± 6.38
	Very often	30	15.20 ± 4.03	9.50 ± 2.54	14.96 ± 2.17	39.66 ± 5.37
	F/p		0.874/0.420	1.056/0.351	**4.455/0.014**	2.645/0.075
Consider Leaving Job	Yes	18	14.72 ± 4.45	8.94 ± 2.85	14.38 ± 2.54	38.05 ± 6.69
	Sometimes	47	15.23 ± 3.28	9.34 ± 2.16	14.68 ± 2.04	39.25 ± 4.76
	No	57	15.70 ± 4.09	8.59 ± 2.55	14.17 ± 2.81	36.47 ± 7.37
	F/p		2.086/0.129	1.176/0.312	0.525/0.593	2.466/0.89
Work Schedule	Day shift	34	14.38 ± 3.50	9.11 ± 2.19	14.61 ± 2.30	38.11 ± 5.38
	Night shift	88	14.46 ± 4.05	8.86 ± 2.56	14.31 ± 2.57	37.64 ± 6.85
	t/p		-0.106/916	0.509/0.612	0.593/0.554	0.359/0.720

X ± SD = Mean and Standard Deviation, **F**: Anova test, **t**: Student t-test, *p*<0.05

Correlation analysis revealed a strong positive correlation between the SCSPI total score and its spiritual care (*r* = 0.853, *p* < 0.001) and individualized care subscales (*r* = 0.824, *p* < 0.001), and a moderate positive correlation with the religiosity subscale (*r* = 0.443, *p* < 0.001). Regarding compassion fatigue, low and non-significant correlations were observed with the spiritual care (*r* = 0.055, *p* = 0.547) and individualized care subscales (*r* = 0.065, *p* = 0.480). A low but statistically significant positive correlation was found between compassion fatigue and the individual religiosity subscale (*r* = 0.200, *p* = 0.027) (Table 3), while no statistically significant relationships were observed between compassion fatigue and the SCSPI total score or the other subscales. These findings suggest that religiosity may function differently from other dimensions of spiritual care perception, potentially reflecting a personal coping orientation rather than a professional care competence. This situation may be related to the use of religiosity as a coping strategy in response to occupational stress.

**Table 3:** Distribution of total score averages between the compassion fatigue scale and spirituality and spiritual care perception scale and its subscales

		1	2	3	4	5
Spirituality and Spiritual Care Subscale (1)	^*^ *r*					
	^**^ *p*					
Individual Care Subscale (2)	^*^r	0.647				
	^**^p	0.000				
Religiosity Subscale (3)	^*^r	0.010	0.135			
	^**^p	0.916	0.139			
SCSPI total score (4)	^*^r	0.853	0.824	0.443^*^		
	^**^p	0.000	0.000	0.000		
CFA total score (5)	^*^r	0.055	0.065	0.200	0.135	

**p < 0.05, *r = Pearson Correlation

## Discussion

This study examined the relationship between compassion fatigue, spirituality, and spiritual care among intensive care nurses. Although each of these concepts has been studied individually, research addressing them together within intensive care settings is scarce. Prior work generally focuses on either compassion fatigue or spirituality and spiritual care, leaving their combined interaction largely unexplored. This gap underscores the contribution of the present study. Examining these variables concurrently may provide insight into how emotional strain and internal meaning-oriented resources coexist in intensive care nursing practice. Moreover, much of the existing evidence comes from emergency or palliative care settings, which may limit its relevance to intensive care nurses. Cultural, institutional, and workload-related differences further constrain comparability across studies; therefore, these contextual factors were considered when interpreting the literature. Given the sustained exposure to critically ill patients in intensive care units, such contextual considerations are particularly relevant when interpreting psychosocial outcomes.

The average age of the participating nurses was 30.49 ± 6.30 years, and their mean duration of work in intensive care units was 5.24 ± 4.18 years. Regarding sociodemographic characteristics, most nurses were female, married, held an undergraduate degree, had 1 to 4 years of experience in intensive care, and worked night shifts. Similar sociodemographic profiles were reported in previous studies, including Amarneh and Al-Dwieb (2022), who studied compassion fatigue and resilience among intensive care nurses; Cho and Cho (2021), who examined compassion fatigue in palliative care nurses; and Borges et al. (2019), who investigated compassion fatigue in emergency intensive care unit nurses [19–21]. This similarity suggests that the sample characteristics are broadly representative of intensive care nursing populations described in the literature, supporting the contextual comparability of the findings. Nevertheless, representativeness does not imply complete generalizability, and cultural and institutional factors should be borne in mind when interpreting these similarities.

Compassion fatigue is known to have numerous negative effects on nurses’ physical and mental health [22]. It may adversely impact healthcare professionals’ performance by causing low self-esteem, job dissatisfaction, difficulties in decision-making, increased intention to quit, and various physical, emotional, and psychological problems [23]. In this study, female nurses, married nurses, those working in neurology intensive care units, nurses with undergraduate and graduate education, nurses dissatisfied with their current units, those wishing to transfer to another unit, nurses with 10–15 years of work experience, nurses intending to leave due to intensive care-related factors, and nurses working daytime shifts demonstrated higher levels of compassion fatigue. The difference in total scores on the compassion fatigue assessment among these groups was statistically significant (*p*< 0.05) (Table 1). Alharbi et al. (2020) reported that higher education levels increased the risk of compassion fatigue among intensive care nurses [8]. This is a point of contention in the literature; while higher education typically enhances coping skills, in the ICU context, it may instead increase clinical awareness and empathy, thereby heightening emotional vulnerability. This suggests that the “educational burden” in ICUs might be related to higher expectations and complex decision-making roles placed on more qualified nurses. Similarly, Aslan and Baykara Mat (2024) found that compassion fatigue was higher in female nurses compared to males [24]. Another study also revealed significantly greater compassion fatigue in women than in men [2]. Borges et al. (2019) reported that female emergency intensive care unit nurses experienced higher levels of compassion fatigue than males [21]. The higher fatigue observed in female and married nurses suggests a “double burden” of caregiving, where professional emotional labor is compounded by domestic responsibilities. While our findings align with Jarrad et al. (2018), they contrast with some literature suggesting that gender differences in CFA disappear when work hours and support systems are controlled, indicating that the impact of gender may be culturally or structurally mediated [25]. Furthermore, Karaca and Aydın Özkan (2024) observed that compassion fatigue increased with nurses’ years of work experience [26]. Pérez-García et al. (2021) identified consequences of compassion fatigue in nurses, including the desire to leave the profession, reluctance to go to work, avoidance of patient care during certain periods due to depression, emotional exhaustion, and lack of motivation [27]. Gustafsson and Hemberg (2022) also found that some nurses considered changing workplaces or leaving the profession because of compassion fatigue [28]. Overall, the findings show that compassion fatigue levels differ across demographic and work-related characteristics among intensive care nurses. These differences are consistent with previous studies and indicate varying risk patterns within intensive care settings; however, they should be interpreted as associative rather than causal, as factors such as education and unit type may reflect differences in workload intensity and moral distress in the ICU environment.

The literature indicates that various factors influence nurses’ understanding of spirituality and spiritual care. These factors include educational background, the nurse’s own spirituality or religious beliefs, years of experience, age, and the clinical environment, all of which affect their SCSPI [29]. In this study, higher spirituality and spiritual care scores were observed among female nurses, single individuals, those aged 20–29, nurses with a high school education, those working in internal medicine intensive care units, nurses with a strong desire to transfer to another unit, and those working daytime shifts. However, many of these differences did not reach statistical significance, no significant differences were found in the average scores of the SCSPI. Conversely, older nurses, those with postgraduate education, and those with longer intensive care experience had lower spirituality and spiritual care scores, although these differences were not statistically significant. Additionally, a statistically significant association was found between nurses’ satisfaction with their intensive care unit and both the spiritual care subscale and total SCSPI scores (*p*<0.05). The significant association between unit satisfaction and SCSPI scores is particularly important. It suggests that spiritual care competence is not merely an individual trait but is also influenced by the organizational climate. Nurses who feel professionally supported are likely to be more “spiritually available” to their patients, whereas a toxic environment may deplete the internal resources necessary to provide spiritual care. Van Leeuwen and Schep-Akkerman (2015) and Baysal et al. (2024) reported that spiritual care competence may be associated with various individual characteristics, including age, gender, education, religious beliefs, work experience, and perspectives on spirituality [30, 31]. Melhem et al. (2016) found that female nurses exhibited higher levels of spirituality and spiritual care compared to their male counterparts [32]. Atarhim et al. (2019) reported a significant relationship between nurses’ education level and spirituality and spiritual care [33]. Rahman et al. (2021) indicated that nurses’ job satisfaction and the clinical unit they worked in were significantly related to total scores and subscale scores of the SCSPI [34]. This study yielded findings consistent with the literature, suggesting that nurses’ levels of spirituality and spiritual care may be related to various individual and organizational characteristics. Female, single, younger, high school–educated nurses and those working in internal medicine intensive care units tended to have higher scores, although most of these differences were not statistically significant. Therefore, these results should be viewed as exploratory tendencies rather than evidence of causal relationships. However, the significant association between satisfaction with the intensive care unit and both the spiritual care subscale and total SCSPI scores indicates that workplace factors may be important for spiritual care competence. Given the limited statistical significance, the findings should be interpreted cautiously and confirmed in future studies with larger samples and diverse clinical settings.

The literature indicates that increased levels of compassion fatigue among nurses negatively affect their ability to apply knowledge and skills in care practices, leading to poorer patient outcomes and reduced quality of care [8, 22]. In our study, intensive care nurses exhibited a moderate level of compassion fatigue. Beevi et al. (2019) reported a high compassion fatigue level of 75% among intensive care and emergency nurses [35]. Similarly, Korkmaz and Alcan (2024) and Ünlügedik and Akbaş (2023) found moderate compassion fatigue levels among intensive care nurses [6, 36]. Conversely, studies by Aslan et al. (2022) and Özan and Polat (2024) reported low compassion fatigue levels in nurses [2, 24]. These variations in compassion fatigue levels across studies may be attributed to differences in demographic and cultural characteristics of nurses, as well as the clinical settings and hospitals in which they work.

In this study, intensive care nurses’ perception of spirituality and spiritual care was found to be at a moderate level (Table 2). Similarly, Kutlu et al. (2020) reported moderate levels of spirituality and spiritual care perception among intensive care nurses [37]. Rahman et al. (2021) found that the lowest subscale score of the SCSPI was in the religiosity dimension [34]. Yıldırım and Ertem (2022) also reported moderate perceptions of spirituality and spiritual care among nurses [38]. In contrast, Neşe and Geçdi (2024) found high levels of spirituality and spiritual care perception in nurses [14]. Atarhim et al. (2019) reported moderate levels of SCSPI among nurses [33]. Additionally, Baysal et al. (2024) and Herlianita et al. (2018) found that nurses had high perceptions of spiritual care [31, 39]. The variations in spirituality and spiritual care perceptions reported across studies likely reflect differences in methodological factors, such as sample characteristics, measurement tools, and training on spiritual care, as well as contextual factors, including institutional support and patient populations. Our study provides data specific to intensive care nurses, highlighting the need to consider these methodological and contextual differences when interpreting results and comparing findings with existing literature.

A highly significant positive correlation was found between the SCSPI score and both the spiritual care and individual subscales. This strong correlation is expected given the part-whole relationship between the total scale and its sub-dimensions. A moderate significant correlation was observed between the religiosity subscale and the other SCSPI subscales. Regarding the relationship between the two main constructs, it was observed that some correlations between compassion fatigue and SCSPI subscales did not reach statistical significance. Accordingly, only the statistically significant correlations were interpreted, and non-significant correlations were not considered evidence of association. Where significant, the associations were generally at a low level. Notably, a significant but weak association was found between compassion fatigue and the religiosity subscale of the SCSPI (Table 3). Although this finding may appear counterintuitive, it may be explained by the possibility that some nurses experiencing higher levels of compassion fatigue increasingly rely on personal religious beliefs as a coping mechanism. Given the limited strength of this correlation, it should be viewed as an exploratory finding. In high-stress environments such as intensive care units, religiosity may serve as an individual resource for emotional regulation and meaning-making rather than as a protective factor that reduces compassion fatigue. Therefore, higher religiosity scores among nurses with greater compassion fatigue may reflect a reactive or compensatory response to occupational stress, rather than a direct causal relationship. Atarhim et al. (2019) reported a moderate positive correlation between nurses’ perceptions of spirituality and spiritual care [33]. Conversely, Aslan and Baykara Mat (2024) found no statistically significant relationship between compassion fatigue and care behavior scores [24]. Similarly, Korkmaz and Alcan (2024) reported no significant association between compassion fatigue and attitudes toward the caregiving role among intensive care nurses [36]. This relationship suggests that religiosity may serve both as a coping mechanism and as a factor increasing emotional sensitivity to patients’ suffering; however, due to the weak nature of these associations, this relationship should not be interpreted as causal. Rather, the relationship appears complex and potentially bidirectional, highlighting the need for prospective and qualitative research to more clearly elucidate the underlying mechanisms.

### Limitations

As the study was conducted using a cross-sectional design, it only provides a snapshot of the relationship between compassion fatigue and perceptions of spirituality and spiritual care. Therefore, causal relationships cannot be established. The study sample was limited to nurses working in intensive care units in Turkey, which may limit the generalizability of the findings to nurses in other clinical settings or countries. The spiritual and religious beliefs of participants may vary regionally within Turkey and influence responses. These cultural factors may limit the transferability of findings to different cultural or religious populations; however, they may contribute to the process of generalization.

### Implications and recommendationsfor practice

As a result of the study, it was observed that intensive care nurses experienced compassion fatigue and that this was associated with their perceptions of spirituality and spiritual care. The results obtained are parallel to similar studies. However, because the study has a cross-sectional and non-interventional design, the findings do not permit causal inference or direct evaluation of specific practice interventions. For this reason, suggestions such as training on spiritual care, strategies for coping with compassion fatigue, or institutional initiatives including spiritual counseling services should be regarded as potential approaches to be examined in future research, rather than direct evidence-based practice recommendations arising from this study. Future longitudinal and interventional studies are needed to determine whether such initiatives effectively reduce compassion fatigue or enhance nurses’ perceptions of spirituality and spiritual care.

## Conclusion

This study demonstrated that intensive care nurses experienced moderate levels of compassion fatigue and moderate perceptions of spirituality and spiritual care. Directly aligned with the study’s aim, the results highlight that demographic and professional factors specifically gender, advanced education, and unit type are significant predictors of emotional exhaustion. The findings suggest that compassion fatigue may be weakly associated with certain dimensions of spiritual perception, particularly religiosity, although the strength of these relationships was limited. The results highlight the potential importance of supporting nurses’ psychological well-being and holistic care awareness in intensive care settings. Workplace-based support strategies, nursing education programs focusing on spiritual care, and institutional policies promoting staff well-being may contribute to improving nursing practice in high-stress clinical environments. As a cross-sectional study, the findings should be interpreted as exploratory and cannot establish causality. Future longitudinal and intervention studies are recommended to further investigate the complex relationship between compassion fatigue, spirituality, and spiritual care perception among intensive care nurses.

## Data Availability

Not applicable.
